# Perfunctory Exercises in Schools

**Published:** 1914-05-30

**Authors:** 


### PERFUNCTORY EXERCISES IN SCHOOLS.

### The Inner Problem of Organised Games.

One of the subjects to which the school doctor is expected to devote some attention is physical exercise. Recently the teaching profession has been much concerned about the strictures which the Board of Education has passed upon the manner in which such exercises are taught in the schools. It will be remembered that some years ago the Board issued a small book, or manual, on the subject. This gave a syllabus of exercises which was recommended for general adoption, and at the time the manual was cordially welcomed, and great expectations were held as to the future of the method which it endorsed. Experience has since shown that these expectations have been rather too high. From the teacher's point of view the book has been criticised mainly on two points. One, that the exercises as described are largely objectless; they are looked upon as a series of set lessons which are performed, more or less correctly, as a matter of routine; the children have no interest in their performance; they do not realise what results are to be obtained from them, and they look upon the time spent in performing them as so much time wasted. The other is that they replace an older system of exercises which had its admitted good points, although it admittedly needed modification in details. From the doctor's point of view both these objections may be supported, and perhaps one or two additional objections may be formulated.

With reference to the objectless character of the exercises in the Code, an objection on this ground may also be laid against the organised games which are now, presumably, so popular in certain schools. Play, after all, is the result of a driving force in the child himself; an organised game is a euphemism for a laboriously evolved series of evolutions which may or may not fascinate the child, and evoke in him a desire to participate in them; it wholly lacks the incentive stimulus that makes the so-called unorganised game so alluring to the child. It is certainly rather remarkable that these organised games taught at the schools have so far found little root in the life of the children outside the school playgrounds, or even in them when the teachers are not present, in strong contrast to certain other school acquirements—for example, Morris dancing—which are honoured outside the school as well as in it. But even an organised game has some object, or at least one that the mind of the child can grasp and appreciate, although it is perhaps true that such grasp only brings the realisation of the fact that it is hardly "the kind of game one would play" if one had a choice in the matter. With the exercises it is wholly different. The child can, and does (as witness the classes under the old School Board *régime*), appreciate the importance of keeping step, marching in formation, holding shoulders and head erect, standing at attention, and so on. He has seen these movements performed by what, when all is said and done, after all appeals very strongly to every child—a military unit, uniformed, marching down the street. But he cannot, and does not, appreciate the object in these Swedish movements when they are employed as part of the routine lessons of the school. He relegates them, then, to that group of subjects in the time-table for which he has the most profound contempt, although he is popularly supposed to love them. Nature-study, strange as it may seem, is one of these subjects, perhaps, just because it is taught, frequently at least, without an attempt to stir the imaginative faculty in the child. Girls, perhaps, look less askance upon these exercises than do boys; they instinctively feel that certain positions in the drill are "dance positions," and they realise that one of the objects of the syllabus is to make their joints supple and give play to their limbs. To the boys, used as they are to the freer and more sensible limb play in their daily out-door games, these perfunctory movements are so much "piffle." This accounts largely for the slack, haphazard way in which the exercises are performed in schools, and for the strictures which the Board's inspectors from time to time pass, unjustly, upon the methods of teaching the exercises. The fault, if there be a fault, lies not in the teachers, but in the syllabus and in the objectless succession of movements which are dignified by the name of physical exercises. The older system, in which a semi-military form of ceremonial drill was used, seems to be, from a psychological point of view, far preferable to the new Code.

From a purely medical point of view the Code may be criticised on several points. One is that the initial position illustrated, and adopted by those who systematically follow the manual, for the standing pose, is a position of strain upon the child which entails a considerable amount of fatigue upon the muscles of the leg and abdomen. The natural position when standing at attention is with the toes pointing forwards, not directly outwards as the manual would have us believe. The latter position alters the pressure points in the foot, especially in the ankle and instep, with the result that a strain is thrown on the flexor tendons of the foot and upon the plantar fascia. The natural walking position is with the foot proceeding straight forward, toes pointing forwards, and the sole of the foot coming to rest flat on the ground when the other leg swings forward; in most of the photographs in the manual it is clear that the child puts his foot forward in a position either of inversion or eversion. That this is no mere academic objection is shown in the fact that a continuance of these exercises with that eversion of the foot leads to flat feet—a condition which is lamentably common in school children—and afterwards to scoliosis. Tannin impressions taken from the foot of a child before starting the exercises and six months later, when he had been doing these exercises more or less regularly, showed that there was a marked flattening of the arch which it was reasonable to ascribe to the strain of this pose, since during the holidays, when no exercises were done, the flattening was lessened. Where there is already a tendency towards flattening of the arch these exercises will undoubtedly tend to accentuate it.

The lunging positions described in the manual are similarly objectionable. The proper lunge needs for its due execution the balance of the hinder part of the body, plus the weight of the arm; at its best it is seen in the lunge with the foil, in its less excellent form in the " point " with a cutlass. The lunge illustrated in the manual is not a lunge at all, but merely a stretch in which the upper arms do not balance but incommode the head and trunk; in a girl it may be a suitable exercise to correct a displacement of the pelvic organs, in which case it would be more properly substituted by the " throw " in basket-ball; in a boy it serves no purpose which is not far more usefully served by the various movements in football or the high catch in cricket. As a matter of fact not one of these elaborate evolutions appears to serve any purpose which is not equally well served by the manifold movements in the swimming lessons which nearly all classes now indulge in. It must not be forgotten that Swedish exercises are in a sense remedial gymnastics, evolved in the first place to correct certain faulty positions in adult patients who are not in the habit of playing games. The school child does a vast variety of evolutions spontaneously, but nearly always with a definite object in view, although he may be, and usually is, quite unconscious of the fact that he employs a particular series of movements for a particular object. To tabulate these movements and make him go through them as a matter of daily routine is to cause, in the vast majority of cases, a certain amount of mental and physical fatigue which is directly detrimental, and wholly destroys whatever good these exercises may do.

In an abnormal child, whose posture or attitude is faulty, many of these exercises may be of excellent service, but in that case they demand modification. For instance, there is no series of exercises in the manual that will enable the teacher to correct cases of scoliosis in pupils who may suffer from slight forms of this spinal deformity. Yet scoliosis is a very common defect in school children, and it should be easy to formulate a small series of special exercises that are suitable for these children, especially in the lower forms which are not taken to the swimming baths. In that case the exercises will possess a definite object which the pupils and teachers will realise and attend to. At present they represent nothing but the office-made ideals of what ceremonial drill should be like. As a basis for future compilation they are excellent; in their proper place—that is in a system of semi-military drill—many of the exercises will serve admirably. In fact, the Australian authorities have already adopted the manual's best points and grafted it to the military drill which is taught in the schools by instructors of the Defence Force with the most excellent results. If something similar can be done here, both the pupils and the teachers will doubtless be grateful for it, while the school doctor will give the manual a more cordial endorsement and approval than he can honestly give at present.

